# Impact of Sugar-Sweetened Beverage Taxation Scenarios in Germany: A Comparative Modelling Study

**DOI:** 10.1093/eurpub/ckac129.340

**Published:** 2022-10-25

**Authors:** KMF Emmert-Fees, A Felea, C Kypridemos, P Scarborough, M Laxy

**Affiliations:** 1 Institute of Health Economics and Health Care, Helmholtz Zentrum München, Neuherberg, Germany; 2 Department of Sport and Health Sciences, Technical University of Munich, Munich, Germany; 3 Department of Public Health, Policy and Systems, University of Liverpool, Liverpool, UK; 4 Nuffield Department of Population Health, University of Oxford, Oxford, UK

## Abstract

**Objectives:**

Taxing sugar-sweetened beverages (SSBs) is recommended to reduce the burden of obesity, cardiovascular disease (CVD) and type 2 diabetes (T2DM). The objective of this study was to quantify the potential long-term health and economic impact of different SSBs taxation scenarios in Germany using simulation models with different granularity.

**Methods:**

We used a multi-state life table Markov cohort (MSLT) and a microsimulation (IMPACT) model to simulate the impact of different SSB taxation assumptions and scenarios on CVD and T2DM in the German population aged 20 years and older over 20 years. Data sources included official population counts, anthropometric and dietary intake data from national surveys and published meta-analyses. Change in beverage consumption by sex and age under different taxation scenarios was estimated using de-novo national price elasticities and changes in body weight were based on an energy equilibrium model. We projected incremental disability-adjusted life years (DALYs) and healthcare costs, comparing results between both models.

**Results:**

Preliminary results from the MSLT model show that a 20% SSB excise tax in Germany could lead to moderate body weight reductions across all age-sex groups. Over 20 years, the tax would reduce healthcare costs by €753 million [95% CI: 527; 1,021] and save 24,380 DALYs [18,460; 33,900] from T2DM alone. Health and economic gains largely depend on the relevance of substitution effects, tax amount and taxed beverage categories. Results including CVD and comparisons with the IMPACT model are work-in-progress.

**Conclusions:**

We show that SSBs taxation in Germany has the potential to reduce healthcare costs and improve population health. The impact of any SSB tax depends on its implementation scenario, which behavioural assumptions hold, and the obesity-related disease outcomes considered for its evaluation. Further research should compare various obesity prevention approaches to support health policy priority setting.

**Key messages:**

• Taxation of sugar-sweetened beverages can reduce healthcare costs and improve population health in Germany.

• The estimated health and economic impact of sugar-sweetened beverage taxation critically depends on tax design and behavioural modelling assumptions.

